# Cross-Scale Correlation Analysis Between Forming Quality and Microstructural Response During SPIF of the Al 1060 Based on PEEQ-Based SSD Density Inference and EBSD Characterization

**DOI:** 10.3390/ma19143152

**Published:** 2026-07-22

**Authors:** Xinyue Zhang, Xiaojing Zhu, Yuhuai Wang, Yaokun Ye, Mingyan Zhao, Teng Zhou, Wenxun Li

**Affiliations:** 1 School of Mechanical & Electrical Engineering, China Jiliang University, Hangzhou 310018, China; p24010855045@cjlu.edu.cn (X.Z.); zhaomingyan@cjlu.edu.cn (M.Z.); 23a0104209@cjlu.edu.cn (T.Z.); p24010855026@cjlu.edu.cn (W.L.); 2 School of Engineering, Hangzhou Normal University, Hangzhou 310018, China; 3 Sinofork Equipment Company Ltd., Huzhou 313300, China; yeyaokun_zl@163.com

**Keywords:** SPIF, forming quality, Kocks–Mecking model, statistically stored dislocation, macro–micro correlation, EBSD

## Abstract

Single-point incremental forming (SPIF) produces localized plastic deformation, resulting in thickness reduction, geometrical deviation, and microstructural evolution. To establish the relationship between forming quality and microstructural response, this study develops a cross-scale analysis framework integrating finite element simulation, Kocks–Mecking (K–M)-based statistically stored dislocation (SSD) density inference, metallographic observation and electron backscatter diffraction (EBSD) characterization. Applied to an Al 1060 truncated-cone part, the framework converts the simulated equivalent plastic strain (PEEQ) into SSD density via the K–M model calibrated using the Voce hardening model and the Taylor relation. The inferred SSD density distribution is then spatially correlated with thinning rate, geometrical deviation, grain size, grain-boundary misorientation, kernel average misorientation (KAM), and geometrically necessary dislocation (GND) density across different forming regions. The inferred SSD density rapidly approached a saturation level of 1.55 × 10^13^ m^−2^, while the high-SSD density region progressively expanded during forming. This regional evolution was qualitatively consistent with the EBSD observations. The average grain size decreased from 30.4 μm to 21.9 μm, and the medium-angle grain-boundary fraction increased from 10.3% to 34.5%. Regionally, thickness reduction correlates strongly with PEEQ accumulation, SSD storage, and grain refinement, whereas geometrical deviation is more closely related to early-stage deformation heterogeneity. These findings provide a physically based route for predicting and controlling SPIF accuracy.

## 1. Introduction

Single-point incremental forming (SPIF) is a highly flexible sheet metal forming process that does not require dedicated dies and is particularly suitable for small-batch, customized, and geometrically complex thin-walled components [1,2]. During SPIF, the forming tool subjects the sheet to repeated localized loading. This process combines in-plane stretching, local and reverse bending, through-thickness shear, and cyclic contact loading [3,4]. This multi-mode coupling causes the material to undergo non-uniform and complex stress paths. On the one hand, this induces typical macroscopic forming quality defects such as local thinning, geometrical deviation, springback, and pillow-like errors [5,6,7]. On the other hand, it drives microstructural evolution, including dislocation accumulation and rearrangement, gradual grain-orientation rotation, and grain-boundary evolution to texture development [8,9,10]. SPIF forming quality is governed by heterogeneous plastic flow under complex stress states. Macroscopic defects represent its external manifestation, whereas microstructural evolution reflects the underlying material response. To reveal the above cross-scale correlation mechanism, Al 1060 was selected as the model material in this paper due to its high stacking fault energy and pronounced deformation-induced microstructural evolution during deformation. Meanwhile, its low density, high plasticity, excellent thermal conductivity and corrosion resistance also make it promising for lightweight thin-walled components [11,12]. Therefore, investigating the coupled macro–micro evolution of forming quality during SPIF of Al 1060 is fundamental to understanding its deformation nature and optimizing process performance.

To obtain the microstructural response during SPIF, optical microscopy (OM), scanning electron microscopy (SEM), and electron backscatter diffraction (EBSD) have been widely employed to characterize formed microstructures. Hussain et al. [11] examined the microstructures of AA5754 and AA6061 sheets before and after forming. They found that wall angle, feed rate, and spindle speed affected grain elongation in AA5754 and the elongation of second-phase particles in AA6061. Ghaferi et al. [12] analyzed the microstructural changes of AA6061 sheets under different heat treatment routes using OM and SEM, clarifying the relationship between heat treatment condition, formability, and hardness evolution. Compared with OM and SEM, EBSD enables quantitative, orientation-resolved characterization of formed microstructures. Shrivastava et al. [9] used EBSD and X-ray diffraction to examine AA1050-H14 sheets during SPIF, enabling the measurement of grain-size variation, crystallographic orientation distribution, and texture evolution at different deformation stages. Kumar et al. [10] further performed high-resolution EBSD on an AA1050-H14 formed part and quantitatively evaluated grain elongation, fragmentation, refinement, low-angle and high-angle grain-boundary formation, and shear-texture development in high-strain regions. Beyond morphological and textural characterization, EBSD orientation data can be used to derive KAM and GND density. These indicators describe local misorientation, lattice curvature associated with orientation gradients, and heterogeneous plastic deformation. Wright et al. [13] reviewed EBSD-based strain analysis and demonstrated the use of orientation data for evaluating deformation-induced misorientation. Kamaya et al. [14] used EBSD-derived misorientation measurement to assess plastic strain in polycrystalline materials at the microstructural scale. Konijnenberg et al. [15] assessed GND densities derived from three-dimensional EBSD data and emphasized the influence of measurement and reconstruction procedures on GND density evaluation. Baudin et al. [16] further discussed dislocation estimation from EBSD measurements and highlighted the sensitivity of EBSD-derived metrics to step size, orientation noise, and data-processing parameters. However, although EBSD can characterize the final spatial distribution of these deformation-related microstructural features, it cannot directly capture the transient accumulation of strain and statistically stored dislocation (SSD) during SPIF. Thus, linking experimental characterization with numerical methods is essential for establishing the full spatiotemporal coupling between the macroscopic deformation field and the microstructural response.

To this end, recent studies have bridged process-scale deformation histories and microstructural evolution by coupling finite element (FE) simulations with crystal plasticity [17], viscoplastic self-consistent (VPSC) [18], surrogate, or data-driven models. Tran et al. [19] combined experimental results with crystal plasticity simulations to analyze microstructure-informed forming limits and local deformation behavior of aluminum alloys. Rakshit et al. [20] combined FE simulation with the VPSC model to transfer macroscopic strain paths to the grain scale, enabling the prediction of micro-texture evolution and anisotropy development during SPIF of an Al-Li alloy. Weeks and Stebner [21] used machine learning surrogate models for crystal plasticity to develop efficient multiscale simulations of incremental sheet forming, improving computational efficiency while preserving a physics-based description of grain-scale microstructural evolution. Harfoush et al. [22] proposed a generative adversarial network-based framework for predicting grain morphology during incremental sheet metal forming. These studies show that microstructure-oriented numerical methods can establish mechanistic links between macroscopic deformation paths and microstructural evolution, including local deformation behavior, texture evolution, anisotropy development, and morphology evolution. Recent studies have further emphasized the role of localized plastic deformation in the microstructural evolution of aluminum alloys during incremental forming. Zhang et al. [23] combined SPIF experiments with TEM characterization and showed that plastic deformation and phase evolution jointly affected dislocation accumulation and mechanical properties in AA6061. Yang et al. [24] used XRD, EBSD, and TEM to reveal dense dislocation structures and their interaction with precipitates during friction-stir-assisted incremental forming of AA5052. However, these methods require detailed material parameters and initial microstructural information and have a high computational cost, limiting their applicability in routine analysis. Therefore, a more practical framework that can link macroscopic deformation with dislocation evolution without requiring extensive microstructure input is highly desirable.

To meet the practical framework requirement of linking plastic deformation with microstructural evolution, various dislocation-based models have been developed. These include the Kubin–Estrin model [25], the three-internal-variable model [26], dislocation-based crystal plasticity models [27], and the Kocks–Mecking (K–M) model [28]. Among these, the K–M model has become one of the widely used work hardening models due to its ability to describe the accumulation and dynamic recovery of SSD with increasing plastic strain through a compact storage-recovery formula [29,30]. The applicability of this model has been validated under various deformation conditions. Wang et al. [31] quantified the effects of shearable clusters and precipitates on dynamic recovery in Al alloys using this model. Balaji et al. [32] coupled a modified Kocks–Mecking–Estrin dislocation model with FE simulation to evaluate substructural changes and dislocation evolution during stress relaxation of AA7075. Akhondzadeh et al. [33] developed a dislocation-based plasticity model using discrete dislocation dynamics data, extending the K–M law to slip-system-level dislocation multiplication. These studies demonstrate the potential of the K–M model for describing dislocation storage, recovery, and strain-driven dislocation evolution. However, its application to estimating SSD evolution from FE-derived plastic strain fields during SPIF of aluminum alloys remains limited.

**Figure 1:**
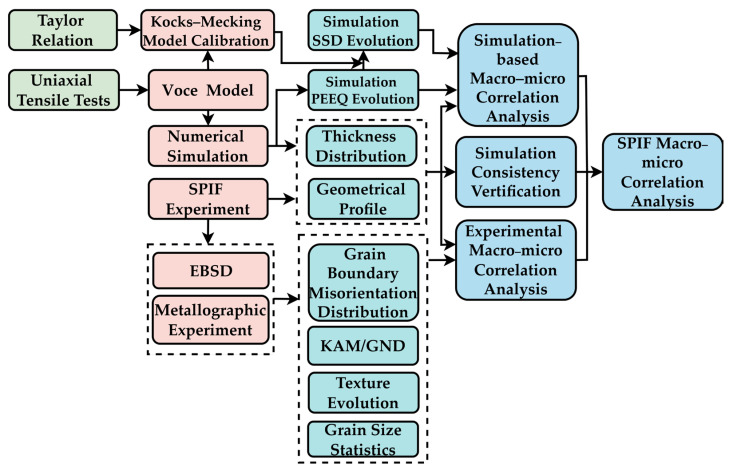
The macro–micro correlation mechanism analysis framework for SPIF forming quality.

Previous SPIF studies have mainly focused on macroscopic forming responses, while the cross-scale relationship between deformation history, dislocation evolution, and microstructural characteristics remains insufficiently understood. In order to gain a deeper understanding of the macro–micro cross-scale evolution of forming quality in SPIF, this study establishes a K–M model-based cross-scale correlation analysis framework for SPIF forming quality, as shown in Figure 1. The framework integrates FE simulation, SSD inference based on the K–M model, and microstructural characterization, and is applied to the SPIF process of an Al 1060 truncated-cone part to reveal the cross-scale evolution mechanism of forming quality. This framework is designed to establish interpretable relationships between strain accumulation, dislocation evolution, microstructural heterogeneity, and forming accuracy. It also provides a microstructure-informed basis for identifying critical deformation regions, assessing FE predictions, and optimizing process parameters.

As illustrated in Figure 1, the framework comprises four stages. First, at the macroscopic level, the geometrical profile and thickness distribution of the experimentally formed truncated-cone part were measured to quantitatively characterize the forming quality. Second, the Voce hardening model was calibrated using the stress–strain data obtained from tensile tests of the Al 1060 sheet, and the parameters of the K–M model were calibrated by combining the Voce hardening law with the Taylor relation. Subsequently, by coupling the equivalent plastic strain (PEEQ) field extracted from the FE simulation, a spatiotemporal evolution model of SSD at different forming stages was established. Third, EBSD and metallographic analyses were used to characterize the microstructural evolution at different deformation stages. The evaluated features included grain morphology, low- and high-angle grain-boundary distributions, crystallographic orientation, and texture evolution. Meanwhile, KAM and GND density were quantitatively derived from the EBSD data to describe the spatial heterogeneity of local plastic deformation. Finally, the macroscopic measurements, inferred SSD density, and EBSD-derived indicators were compared through spatial registration and regional trend analysis. These comparisons were used to examine the relationships between plastic strain accumulation, dislocation evolution, microstructural heterogeneity, and forming quality.

## 2. Materials and Methods

### 2.1. Al 1060 Aluminum Alloy and Basic Mechanical Properties

Al 1060, widely used in lightweight components, was selected as the research material in this study. Al 1060 is a typical commercially pure aluminum alloy with good ductility, high thermal and electrical conductivity, and good corrosion resistance. These properties make it suitable for investigating the relationship between macroscopic forming quality and microstructural evolution during SPIF.

To obtain the material parameters required for FE simulation and dislocation evolution analysis, uniaxial tensile tests were performed on dog-bone sheet specimens using a computer-controlled universal testing machine (WDW-50C; Shanghai Hualong Test Instruments Co., Ltd., Shanghai, China; accuracy class 0.5) according to ASTM E8/E8M [34]. These tensile specimens were prepared along the rolling direction of the sheet, and the tests were conducted at room temperature. The true stress–strain response was obtained. The Voce hardening model, shown in Equation (1), can describe the hardening behavior of the material over a relatively large strain range [35].

(1) $$\sigma =\sigma_{s}\left(1-Ae^{-B\varepsilon_{p}}\right)$$

where σ is the true stress in the strain-hardening stage, $\sigma_{s}$ denotes the saturation flow stress, $\varepsilon_{p}$ is the plastic strain corresponding to the PEEQ in the FE simulation, and *A* and *B* are Voce hardening parameters in the Voce hardening law: *A* determines the initial offset from the saturation stress, and *B* controls the saturation rate.

The Voce hardening parameters and baseline mechanical properties were determined in our previous study [7]. These mechanical properties and hardening parameters were directly used as material parameters in the subsequent K–M dislocation evolution analysis and FE simulation.

### 2.2. Kocks–Mecking Dislocation Evolution Model Based on Voce Parameters

In this study, the K–M model was used to characterize the evolution of SSD with PEEQ [28,30]. The model considers both dislocation storage induced by plastic deformation and dislocation annihilation caused by dynamic recovery and can describe the transition of the dislocation density from rapid growth to gradual stabilization during continuous plastic shear deformation. Its basic form is expressed as

(2) $$\frac{d\rho }{d\gamma }=\left(\frac{k_{1}}{b}\sqrt{\rho }-k_{2}\rho \right)$$

where ρ denotes the SSD density, γ denotes the equivalent shear strain, *b* is the magnitude of the Burgers vector, $k_{1}$ and $k_{2}$ are the dislocation storage and dynamic recovery coefficients, respectively. A larger $k_{1}$ indicates stronger dislocation multiplication during plastic deformation, whereas a larger $k_{2}$ indicates more significant dislocation annihilation, rearrangement, and dynamic recovery.

To use the PEEQ obtained from the FE model, the equivalent shear strain was related to the PEEQ by

(3) $$d\gamma =Md\varepsilon_{p}$$

where *M* is the Taylor factor. Substituting Equation (3) into Equation (2), the K–M relationship can be solved and written as

(4) $$\sqrt{\rho }=\frac{k_{1}}{bk_{2}}-\left(\frac{k_{1}}{bk_{2}}-\sqrt{\rho_{0}}\right)\exp\left(-\frac{Mk_{2}}{2}\varepsilon_{p}\right)$$

where $\rho_{0}$ denotes the initial SSD and b denotes the magnitude of the Burgers vector. To solve the coefficients, $k_{1}$ and $k_{2}$, of the K–M model in Equation (2), considering the form of the Voce hardening model in Equation (1), the Taylor relation was introduced [36]:

(5) $$\sigma =\sigma_{s}+M\alpha Gb\sqrt{\rho }$$

where α is the dislocation interaction coefficient and *G* is the shear modulus. These parameters were selected independently according to the crystallographic and mechanical properties of annealed Al 1060 and commonly used assumptions for face-centered cubic (FCC) polycrystals [30,37].

Because orientation-dependent Taylor factors were not calculated for individual grains or deformation regions, the Taylor factor, M, was taken as 3.06, a commonly used effective value for randomly oriented FCC polycrystals [37]. The dislocation interaction coefficient, α, was set to 0.3, which lies within the typical range used for FCC metals and represents the average strength of dislocation interactions [30]. The shear modulus, *G*, was taken as 26 GPa, corresponding to the room-temperature shear modulus of aluminum [38]. This value is consistent with that calculated from the isotropic elastic relation G=E/[2(1+μ)] using mechanical properties listed in a previous study [7]. For FCC aluminum, the Burgers vector of a perfect dislocation lies along the <110> direction and can be expressed as b=a/2<110>, where *a* is the lattice parameter of FCC aluminum [39]. Therefore, its magnitude is $b=a/\sqrt{2}$. Using *a* = 4.05 × 10^−10^ m, b was calculated as 2.86 × 10^−10^ m. These parameters were not treated as arbitrary fitting variables but were selected on the basis of the crystallographic and mechanical properties of FCC aluminum and commonly adopted literature values [30,37,38,39]. Nevertheless, M and α represent spatially averaged effective parameters. Their use may influence the absolute magnitude of the inferred SSD density. Therefore, the present analysis emphasizes the relative spatial distribution and evolution trend rather than the absolute SSD values. Substituting Equation (4) into Equation (5) gives

(6) $$\sigma =\sigma_{s}+M\alpha Gb\left[\frac{k_{1}}{k_{2}}-\left(\frac{k_{1}}{k_{2}}-\sqrt{\rho_{0}}\right)\exp\left(-\frac{Mk_{2}}{2}\varepsilon_{p}\right)\right]$$

where $\rho_{0}$ denotes the initial SSD set to 1.0 × 10^12^ m^−2^, according to the typical order of magnitude for annealed aluminum. Equation (6) has an exponential saturation form that is consistent with the Voce model shown in Equation (1). By comparing Equation (6) with Equation (1), the corresponding coefficients of the K–M model can be established as

(7) $$\left\{\begin{array}{l} k_{1}=\frac{2AB\sigma_{S}}{\alpha M^{2}G}\\ k_{2}=\frac{2B}{M} \end{array}\right.$$

**Figure 2:**
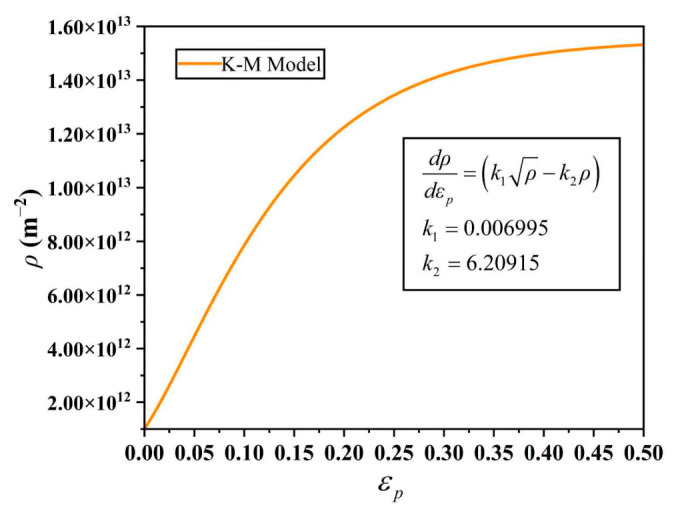
Calibrated result of the K–M model.

Solving Equation (7) yielded $k_{1}$ = 0.006995 and $k_{2}$ = 6.20915. The calibrated result of the K–M model is shown in Figure 2. Figure 2 illustrates that the saturation SSD density, $\rho_{\mathrm{sat}}$, of the material is approximately 1.55 × 10^13^ m^−2^.

After $k_{1}$ and $k_{2}$ were obtained, the PEEQ extracted from the FE model was used as the input plastic strain to calculate the SSD distribution and evolution trend in different forming regions. The K–M model provides a theoretical basis for the subsequent analysis of the correlation between local plastic strain accumulation, dislocation storage, and macroscopic forming quality.

### 2.3. SPIF FE Model and SSD Density Inference

Because of the highly heterogeneous local plastic deformation during SPIF, experimental methods cannot continuously capture the internal strain and stress states of the sheet metal. Therefore, an FE model of the SPIF process for the Al 1060 truncated-cone part was established to obtain the thickness distribution, geometrical profile, and PEEQ field, providing the basis for subsequent SSD inference.

The SPIF process was simulated using Abaqus/Explicit 2021. The sheet was modeled as a deformable body, while the forming tool and clamping platform were treated as rigid bodies. The sheet (200 mm × 200 mm × 1.1 mm) was fully fixed along all edges and discretized using shell elements with local mesh refinement in the forming region. The global mesh size was 2 mm. Mesh refinement generally improves the accuracy of the numerical solution but also increases the computational cost. Based on repeated trial simulations, a mesh size of 2.0 mm was selected because it provided sufficiently stable results for the present analysis while maintaining a reasonable computational time.

**Figure 3:**
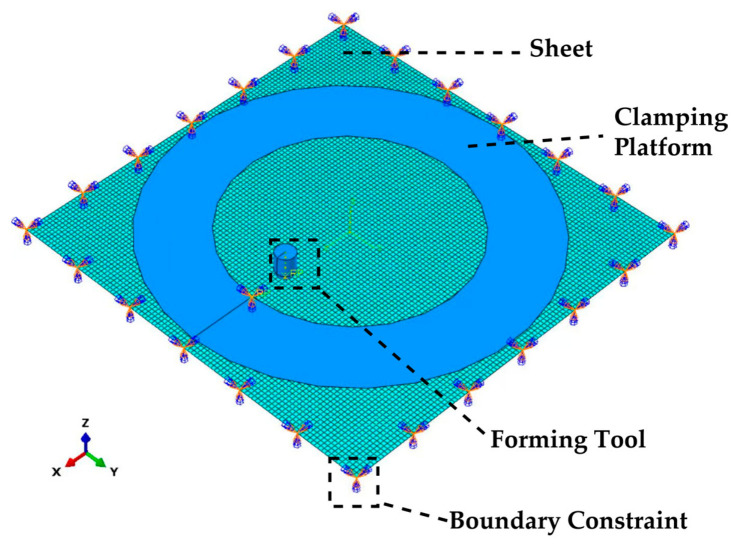
FE mesh model of the Al 1060 sheet for SPIF.

Figure 3 illustrates the FE mesh model. Four-node reduced-integration shell elements (S4R) were primarily used in this model, with three-node elements (S3R) in transition regions. During SPIF, the tool–sheet interface experiences continuous sliding contact, and friction conditions affect material flow, strain localization, and thickness uniformity. Excessive friction may deteriorate surface quality, while appropriate lubrication can improve forming stability. Friction conditions may also influence deformation-induced microstructural evolution through their effects on plastic deformation. The role of lubrication and surface interactions in controlling friction behavior has been reported in previous studies [40]. In this study, lithium-based grease was applied, and a Coulomb friction model with a coefficient of 0.1 was adopted based on previous SPIF simulations of aluminum sheets [41]. Material behavior was defined using the mechanical properties and Voce hardening parameters of Al 1060 described in our previous study [7].

The optimized process parameters obtained in the previous study [7] were adopted as the input conditions, including a tool radius of 4.0 mm, an initial sheet thickness of 1.1 mm, a vertical step-down of 0.19 mm, a forming angle of 30°, and a contour toolpath. The target geometry was specified as a truncated-cone component with a forming depth of 25 mm, an upper radius of 50 mm, and a lower radius of 7 mm.

To assess the reliability of the simulation, the predicted macroscopic responses, including the thickness distribution and geometrical deviation, were compared with the experimental results (see Section 3.1, below, for details). Following this macroscopic validation, the PEEQ fields extracted from the selected representative regions were used as inputs for the K–M model to solve the SSD density distributions. Furthermore, the validated FE model enabled analysis of the relationships between local strain accumulation, dislocation evolution, and macroscopic forming quality.

### 2.4. SPIF Forming Experiment and EBSD Characterization

To obtain macroscopic forming quality data and microstructural responses of the Al 1060 sheet during SPIF, the truncated-cone part was experimentally formed, and EBSD characterization was used to analyze microstructural differences between regions with different forming depths. The macroscopic measurements were used to validate the FE model, whereas the EBSD results provided experimental insight into the SSD density evolution inferred by the K–M model.

**Figure 4:**
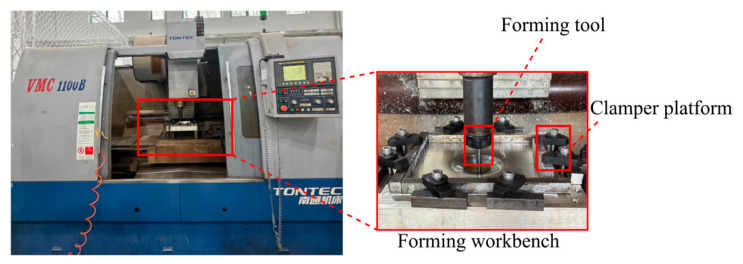
Experimental setup for SPIF.

The SPIF experiment was performed on a VMC1100B computer numerical control machine tool manufactured by Nantong Machine Tool Co., Ltd., Nantong, China, as shown in Figure 4. The experimental conditions were kept identical to those used in the FE model to ensure consistency between simulation and experiment. Lithium-based grease was applied to the tool–sheet interface to reduce friction and improve process stability.

After forming, the formed truncated-cone part was sectioned along the axial symmetry plane using wire electrical discharge machining. The cross-sectional geometrical profile was measured using the HaloScan Performance handheld optical 3D scanner (Hexagon Manufacturing Intelligence, Qingdao, China). The scanner was operated in the standard scanning mode, with a specified accuracy of 0.01 mm and a measurement resolution of 0.01 mm. Before each measurement, the scanner was calibrated according to the manufacturer’s procedure.

The measured profile was registered with the nominal profile using datum-based alignment. At each measurement position, the geometrical deviation was calculated as the axial distance between the measured and nominal profiles. The maximum absolute value was defined as the maximum geometrical deviation. Three replicate forming experiments were conducted, and the measurement results are reported as the mean ± standard deviation. For the thickness measurements, 50 equally spaced locations were selected at 4 mm intervals along the sectioning direction. The local thickness was measured using a micrometer.

Following the SPIF experiment, EBSD characterization was performed on specimens cut from four representative regions. Region 1 was located in the peripheral area outside the tool path and was not contacted by the forming tool. It was therefore designated as the unformed reference region for evaluating deformation-induced microstructural changes. Regions 2, 3, and 4 represented the initial, intermediate, and final forming regions, respectively, and experienced progressively increasing deformation histories. This sampling strategy was intended to identify the main regional microstructural trends.

**Figure 5:**
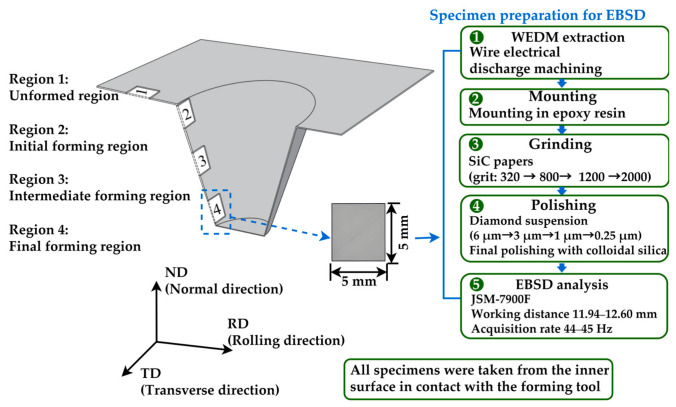
Specimen cutting and preparation for EBSD.

Before EBSD, the rolling direction of the as-received Al 1060 sheet was identified and marked. The rolling direction (RD), transverse direction (TD), and normal direction (ND) were used to define a common sample coordinate system. During EBSD acquisition and data processing, RD and TD were aligned with the X1 and X2 axes of the sample coordinate system, respectively, while ND was normal to the analyzed sheet surface. This coordinate convention was maintained for all four regions to ensure direct comparison of crystallographic orientations. As illustrated in Figure 5, four specimens (5 × 5 mm^2^) were cut from the formed part along the rolling direction using wire electrical discharge machining (WEDM). These specimens were subjected to mounting, grinding, polishing, and etching. The etched microstructures were examined using a field-emission scanning electron microscope (JSM-7900F; JEOL Ltd., Tokyo, Japan). EBSD data were acquired using an Oxford Instruments EBSD system equipped with AZtec software 2.1.

Three independent EBSD fields of view were acquired within each region. Grain size, KAM, and GND density are reported as the mean ± standard deviation. This repeated-field strategy was adopted to reduce the influence of local grain-to-grain heterogeneity and field-of-view selection. To ensure comparability, identical EBSD acquisition parameters were applied, including an accelerating voltage of 20 kV and a step size of 1.0 μm. The scan area was 247 μm × 247 μm, with indexing rates exceeding 80%.

## 3. Results and Discussion

### 3.1. FE Model Validation and Macroscopic Forming Quality Characteristics

The thinning rate and geometrical profile obtained from the experiments and simulations were compared. These two quantities were selected because they directly reflect the accumulated plastic deformation and geometrical accuracy of the formed part, which are the primary macroscopic responses relevant to the subsequent PEEQ extraction and SSD density inference.

**Figure 6:**
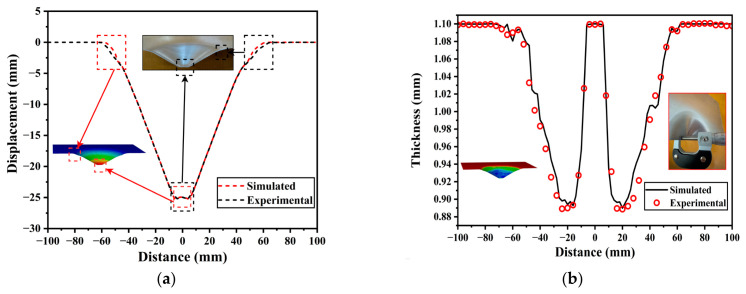
Experimental and simulated forming quality results: (**a**) geometrical profiles and (**b**) thickness distributions.

Figure 6 shows a comparison of experimental and simulated forming quality for the truncated-cone part under the optimized parameter conditions. The experimental part was formed successfully without visible cracking or severe loss of shape stability.

Figure 6a compares the experimental and simulated geometrical profiles. Based on three replicate forming experiments, the maximum geometrical deviation was 2.448 ± 0.02 mm, whereas the simulated value was 2.372 mm, giving a relative difference of 3.10%. The discrepancy between the two profiles was mainly localized near the flange. This localized discrepancy may reflect the combined effects of the idealized boundary condition, downward tool motion, accumulated out-of-plane sheet deformation, and local bending and elastic recovery near the wall–flange transition. Although slight fixture compliance and local sliding may occur during the experiment, the persistence of relatively large localized errors indicates that the boundary condition alone cannot fully explain the discrepancy. The profile comparison also shows a slight out-of-plane deformation in the nominally undeformed bottom region, corresponding to the pillow effect commonly observed in SPIF. This effect is generally associated with the redistribution of residual stresses and elastic recovery in the unsupported bottom region [42,43]. Their reproduction in both the experiment and simulation provides qualitative evidence that the FE model captures the characteristic deformation modes of SPIF.

As shown in Figure 6b, the unformed region maintained an essentially constant thickness, whereas the formed wall and the wall–bottom transition region underwent evident thinning. Based on three replicate forming experiments, the minimum thickness was 0.8887 ± 0.001 mm. The simulated value was 0.8899 mm. The corresponding thinning rates were 19.213 ± 0.072% and 19.101%, respectively. The relative difference in minimum thickness was only 0.14%. Although a thinning rate of approximately 20% is within the expected range for the present geometry and forming conditions, the consistency between the measured and simulated values confirms that the FE model reasonably predicts the magnitude and spatial distribution of sheet thinning.

Accordingly, the macroscopic results in this section are presented as model-validation evidence. The validated model was subsequently used to extract the relative spatial distribution and evolution trend of PEEQ, which provides the strain input for the K–M-based SSD density inference. Nevertheless, the validation was based on macroscopic geometry and thickness rather than direct measurement of the complete internal strain field.

### 3.2. FE-Derived PEEQ Field and K–M-Inferred SSD Density Evolution

**Figure 7:**
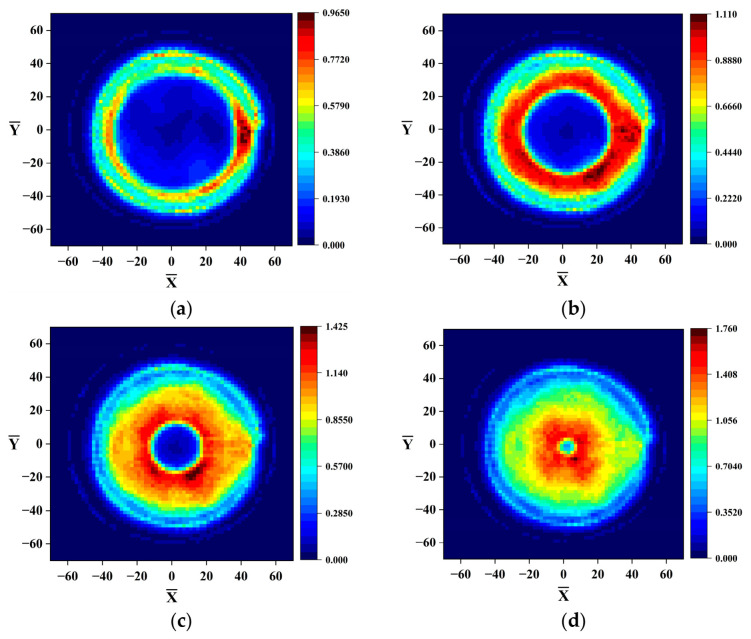
PEEQ contours of the truncated-cone part at different SPIF simulation stages: (**a**) *t* = 0.25 T; (**b**) *t* = 0.5 T; (**c**) *t* = 0.75 T; (**d**) *t* = T.

The simulated PEEQ field was extracted and used as the input for the K–M model to infer SSD density evolution in different forming regions. Figure 7 shows the PEEQ contours at four representative forming stages, including the unformed stage (*t* = 0), initial forming stage (*t* = 0.25 T), intermediate forming stage (*t* = 0.5 T), late forming stage (*t* = 0.75 T), and final forming stage (*t* = T), where T denotes the total forming time. The color bar in Figure 7 represents the magnitude of equivalent plastic strain, which is a dimensionless measure of accumulated plastic deformation. Blue denotes lower accumulated plastic strain, whereas red denotes higher accumulated plastic strain. The average and maximum PEEQ values at different stages are summarized in Table 1. In the unformed stage, the sheet had not yet undergone plastic deformation. In the initial forming stage, PEEQ first increased in the local region where the tool contacted the sheet, with the maximum value reaching 0.965, indicating pronounced plastic deformation in this region. As forming proceeded, the high PEEQ region gradually migrated along the forming path toward the intermediate forming region. The maximum PEEQ increased to 1.110 in the intermediate forming stage and further to 1.425 in the late forming stage. In the final forming stage, the maximum PEEQ reached 1.760, and the high-value region was mainly concentrated in the bottom transition region.

The PEEQ distribution statistics at different stages are also listed in Table 1. As the forming proceeded, the proportion of nodes with PEEQ > 0.5 increased from 3.74% in the initial forming stage to 10.11% in the intermediate forming stage and finally to 14.98%. The proportion of nodes with PEEQ > 1.0 also increased from 1.12% in the intermediate forming stage to 7.53% in the final forming stage. This indicates that plastic strain does not accumulate instantaneously during SPIF in a concentrated manner but continuously accumulates with layer-by-layer tool loading and spatially migrates from the outer wall toward the bottom transition region.

**Figure 8:**
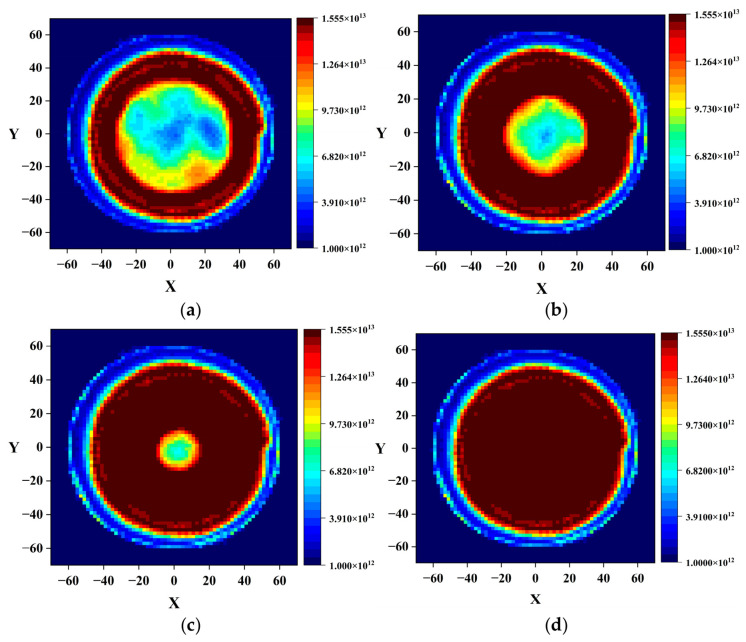
K–M-inferred SSD density contours at different SPIF simulation stages: (**a**) *t* = 0.25 T; (**b**) *t* = 0.5 T; (**c**) *t* = 0.75 T; (**d**) *t* = T.

The PEEQ values at different forming stages were used as the input for the K–M model, and the corresponding SSD density evolution results were obtained. The SSD density contours at different stages are shown in Figure 8, where the color bars represent the magnitude of SSD density, with blue indicating regions of relatively low dislocation density and yellow-to-red colors denoting regions with progressively higher SSD densities. The corresponding statistics are listed in Table 2. As shown in Table 2, the initial SSD density was 1.00 × 10^12^ m^−2^. The average SSD density increased to 4.39 × 10^12^ m^−2^ in the initial forming stage; further increased to 4.60 × 10^12^ m^−2^ and 4.68 × 10^12^ m^−2^ in the intermediate and late forming stages, respectively; and reached 4.75 × 10^12^ m^−2^ in the final forming stage. Although the maximum SSD density approached the saturation value of 1.55 × 10^13^ m^−2^ as early as the initial forming stage, the high SSD density region continued to expand during forming. Specifically, the proportion of nodes with an SSD density >1.0 × 10^13^ m^−2^ increased from 15.19% in the initial forming stage to 19.04% in the intermediate forming stage. It further increased to 20.87% in the late forming stage and 21.26% in the final forming stage. The proportion of nodes with an SSD density >1.5 × 10^13^ m^−2^ increased from 0.66% in the initial forming stage to 6.78% in the intermediate stage, 10.63% in the late forming stage, and 12.19% in the final forming stage. This indicates that the main change in dislocation storage during late forming is no longer a substantial increase in the maximum value, but rather an expansion of the high-dislocation region. These results demonstrate the temporally cumulative nature of SSD evolution. Dislocation storage first appeared in the tool–sheet contact region. As the tool moved along the predefined path, the high-SSD density region expanded with the plastically deformed area and gradually extended toward the bottom transition region.

**Figure 9:**
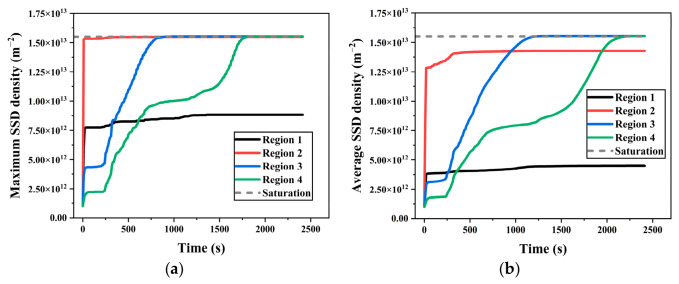
Regional evolution of the K–M-inferred SSD density during SPIF: (**a**) maximum SSD density; (**b**) average SSD density.

To further quantify the temporal evolution of dislocation storage in different regions, the maximum and average SSD densities were extracted from four representative regions during SPIF, as shown in Figure 9. The maximum-SSD density curves in Figure 9a show that the local peak SSD density in region 2 rapidly approached the K–M saturation level, indicating that localized dislocation storage can reach saturation shortly after tool contact. Region 3 exhibited a delayed increase and gradually approached saturation, whereas region 4 showed the latest but most pronounced increase in the late stage. This sequential evolution indicates that the formation of high-SSD density regions follows the tool path and reflects the layer-by-layer accumulation of plastic deformation during SPIF.

As shown in Figure 9b, the average SSD density provides a more stable description of the population within each region. The maximum SSD density reached saturation relatively early. However, the average SSD density continued to increase in regions 3 and 4, indicating progressive expansion of the high-SSD density regions. Therefore, the regional SSD density evolution curves confirm that SSD density increases with accumulated plastic strain and gradually expands from region 2 toward regions 3 and 4.

The PEEQ and SSD results reveal the progressive accumulation and spatial migration of local plastic deformation during SPIF. The high-PEEQ and high-SSD density regions gradually moved and expanded with layer-by-layer tool–sheet contact. Correspondingly, the regional maximum-SSD density evolution curves show that the local maximum SSD density can approach the K–M saturation level early, whereas the average SSD density exhibits a delayed increase. This indicates that in the late forming stage, SPIF is dominated not by a continuous increase in the peak SSD density, but by the progressive expansion of high-dislocation regions. Therefore, the PEEQ-based SSD inference results provide a temporal and spatial basis for explaining regional differences in dislocation storage and subsequent microstructural response during SPIF.

### 3.3. Microstructural Characterization Results

To analyze the microstructural responses of the Al 1060 sheet after SPIF, metallographic observation and EBSD characterization were performed on different regions of the formed truncated-cone part. Metallographic observations were used to examine grain morphology and microstructural distribution. EBSD was used to characterize grain size, grain-boundary misorientation, texture, orientation distribution functions, KAM, and GND density [19]. Although EBSD characterization was performed on the formed part, different regions experienced different deformation histories during SPIF. Therefore, these regional EBSD results can be used to infer the deformation mechanism and microstructural evolution characteristics associated with incremental forming.

#### 3.3.1. Metallographic Grain Morphology and EBSD Grain Size Statistics

**Figure 10:**
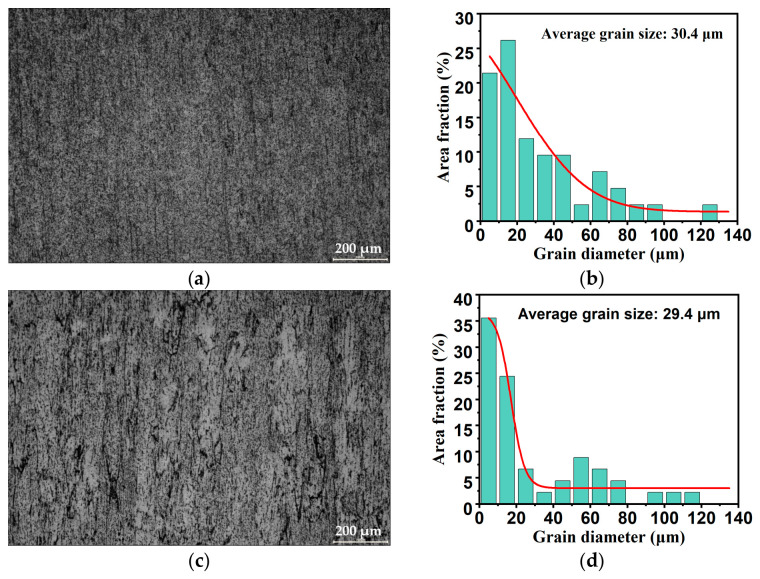

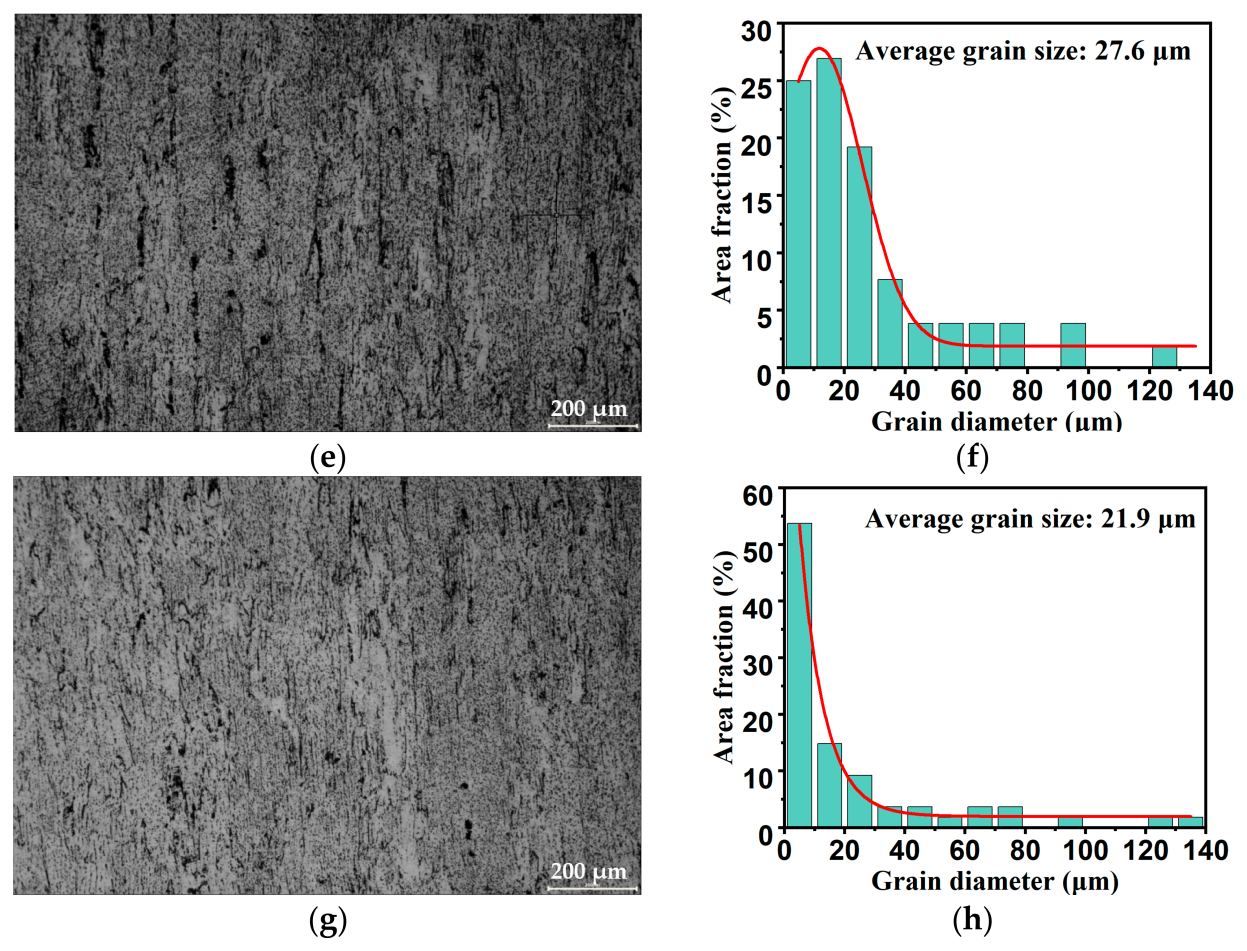
Metallographic grain maps and EBSD grain-size distributions: (**a**,**b**) region 1; (**c**,**d**) region 2; (**e**,**f**) region 3; (**g**,**h**) region 4.

The metallographically etched grain structures of different regions and the grain size statistics obtained from EBSD grain reconstruction are shown in Figure 10. The red line represents the log-normal fitting of the grain size distribution. Region 1 was designated as the unformed reference because it was located outside the tool path and exhibited an FE-predicted PEEQ value of zero. Its microstructure showed relatively intact grain morphology and a uniform grain distribution. The grain morphology and grain-size distributions in regions 2–4 were therefore compared directly with those in region 1. Grain morphology changed progressively with increasing forming depth. Grain refinement and microstructural heterogeneity also became more pronounced, indicating that repeated localized tool loading altered the original microstructure.

From the perspective of grain size, the average grain size of region 1 was 30.4 ± 2.1 μm. With increasing forming depth, the grain size gradually decreased to 29.4 ± 1.5 μm (region 2), 27.6 ± 1.7 μm (region 3), and 21.9 ± 1.4 μm (region 4), showing a clear refinement trend [44]. The progressive reduction in average grain size from region 1 to region 4 is consistent with the microstructural evolution reported for AA1050 sheets subjected to SPIF. Shrivastava and Tandon [9] observed increasingly heterogeneous grain deformation and grain refinement with increasing forming strain, while Kumar et al. [10] reported enhanced grain fragmentation and substructure development in highly strained regions of incrementally formed AA1050 parts. These findings support the interpretation that repeated localized stretching, bending, and shear deformation along the tool path promoted intragranular subdivision and the development of finer deformation structures in the present Al 1060 sheet. This indicates that continuous plastic strain accumulation during forming promoted dislocation multiplication and the formation of subgrain structures within grains. With further strain accumulation, these substructures were progressively subdivided and rearranged through recovery-related processes, resulting in more pronounced grain refinement in region 4. Further analysis of the grain size distribution shows that region 1 exhibited a distinct peak in the 60–70 μm range, whereas region 2 showed a clear peak in the 50–60 μm range, both of which were significantly higher than neighboring size intervals. This feature indicates that a certain proportion of coarse original grains remained in the initial sheet and shallow-deformation region, reflecting that the level of deformation was insufficient to fully fragment the original microstructure. These coarse grains may originate from residual rolled microstructures; in low-strain regions, they experienced only limited deformation and substructural evolution and were not sufficiently refined. As the deformation increased, local strain significantly increased, grain refinement and microstructural reconstruction became more complete, and the peak associated with coarse grains disappeared [45].

#### 3.3.2. EBSD Texture Evolution Characteristics

**Figure 11:**
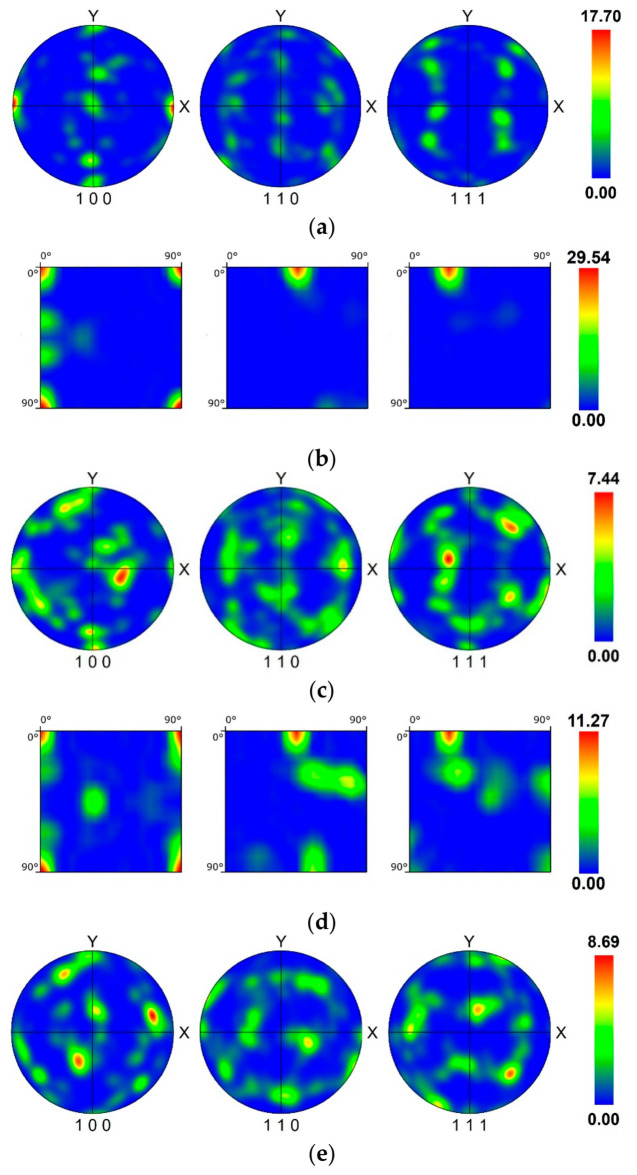

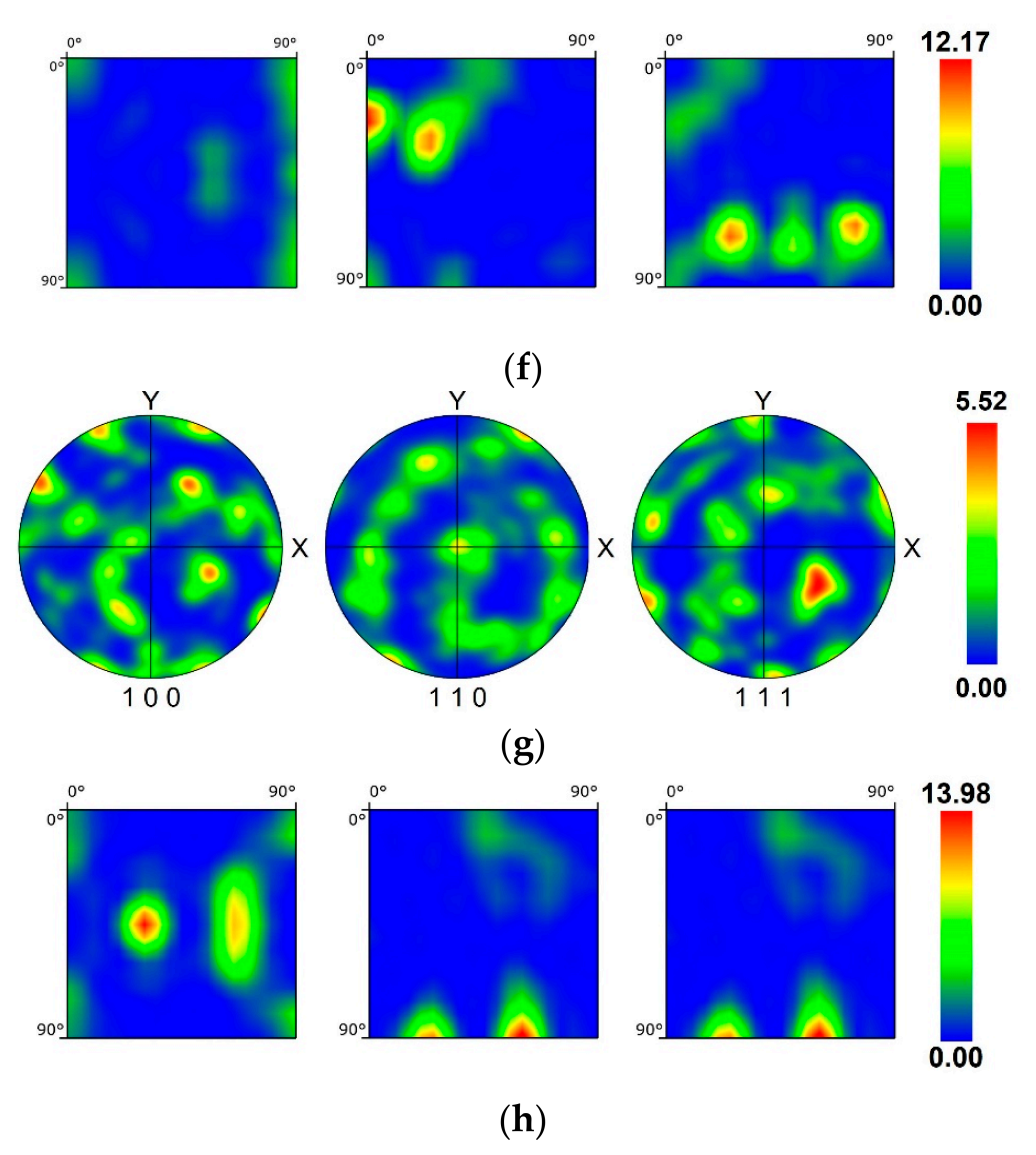
EBSD pole figures and ODF sections: (**a**,**b**) region 1; (**c**,**d**) region 2; (**e**,**f**) region 3; (**g**,**h**) region 4.

Figure 11 presents the EBSD pole figures and ODF analysis results for different forming regions, revealing the texture evolution characteristics during SPIF. In the sample coordinate system used for texture analysis, the X and Y axes shown in the pole figures denote the projections of the macroscopic sample directions on the pole-figure plane. Pole figures mainly characterize crystal orientation distribution, texture intensity, and orientation tilting, whereas ODF results, plotted in Bunge–Euler space $\left(\varphi_{1},\Phi ,\varphi_{2}\right)$, further identify specific texture components such as Cube, Copper, S, P, and Goss. Combining these analyses helps to more comprehensively reveal the grain-orientation rotation and texture reconstruction of the Al 1060 sheet during SPIF [46].

As shown in Figure 11a,b, region 1 exhibited a typical strong Cube texture, mainly with the {001} <100> orientation. The maximum texture intensity was 17.70, indicating a distinct preferred orientation in the original Al 1060 sheet. The ODF results further show that the maximum orientation densities of region 1 at the typical $\varphi_{2}$ = 0°, 45°, and 65° sections were 36.6, 30.1, and 30.1, respectively, with the dominant orientations all corresponding to Cube-related orientations. This indicates that the texture components in the unformed region were relatively concentrated and stable and that the original sheet retained a strong Cube orientation. These results indicate that the texture components in the unformed region were relatively concentrated and stable. Because region 1 was located outside the tool path and exhibited an FE-predicted PEEQ value of zero, its texture characteristics were used as the unformed reference stage. The texture intensity, crystallographic orientation rotation, and newly developed texture components in regions 2–4 were subsequently evaluated relative to this reference. The consistency between the pole-figure and ODF results further indicates that region 1 had not undergone pronounced plastic deformation and retained texture characteristics representative of the unformed reference stage.

Figure 11c,d illustrate that the original Cube texture in region 2 began to weaken, and the texture gradually transformed from a single strong preferred orientation into a tilted sheet texture. The pole-figure results show that region 2 mainly formed a sheet texture in which the {111} plane was parallel to a 10° tilt about the X1 axis (the rolling direction in the sample coordinate system). Its maximum texture intensity was 7.44. A secondary texture was also present in which the {001} plane was parallel to a 10° tilt about the X1 axis with a secondary maximum value of 7.09. Compared with region 1, the texture intensity of this region decreased significantly, indicating that local tool loading had induced grain-orientation rotation and dispersion of the original preferred orientation. The ODF results show that the maximum orientation in the $\varphi_{2}$ = 0° section of region 2 decreased to 13.3 and still corresponded to a Cube-related orientation. In the $\varphi_{2}$ = 45° section, in addition to the Cube component, a Copper {112} <111> texture appeared with an intensity of 7.0. In the $\varphi_{2}$ = 65° section, an S {123} <634> texture appeared with an intensity of 6.6. These results indicate that region 2 still retained some Cube-related orientation, but typical plastic deformation texture components of face-centered cubic metals had begun to appear, suggesting a transition from the original Cube-dominated stage to a multi-component texture stage.

Figure 11e,f show that, in region 3, the texture components became more complex. The pole-figure results show that this region was mainly manifested as a sheet texture in which the {001} plane was parallel to a 15° counterclockwise tilt about the X1 axis, with a maximum texture intensity of 8.69. A secondary sheet texture was also present in which the {111} plane was parallel to a 45° tilt about the X1 axis, with a secondary maximum value of 7.44. These results indicate that, with increasing forming depth, the grain-orientation rotation angle further increased and more obvious competition between multiple orientations occurred. Compared with region 2, the texture maximum value of region 3 increased slightly, indicating that changes in the local strain path and strain direction may have re-strengthened some orientation components. The ODF results further show that the Cube-related texture in region 3 weakened markedly, whereas Copper-related components strengthened. In the $\varphi_{2}$ = 45° section, the maximum orientation was 12.3, corresponding to the Copper {112} <111> texture, and a Copper-related component with an intensity of 10.2 was also present. In the $\varphi_{2}$ = 65° section, the maximum orientation was 10.6, corresponding to a residual Cube-related orientation. Crystal-orientation rotation in region 3 was no longer confined to small deviations from the original Cube orientation. Instead, the texture evolved toward Copper-type orientations, with residual Cube and enhanced Copper components coexisting.

Figure 11g,h illustrate that, in region 4, the overall texture intensity decreased significantly. The dominant texture in region 4 showed a {111} plane tilted by approximately 40° about the X1 axis, with a maximum texture intensity of 5.52. A secondary {001}-related texture was tilted by approximately 30° tilt about the X1 axis, with a maximum value of 4.86. Compared with the previous regions, the texture maximum value in region 4 decreased significantly, indicating that under higher plastic strain, the grain-orientation distribution became more dispersed and the original strong Cube texture was further weakened. The ODF results show that the P texture and Goss {011} <100> texture were significantly enhanced in region 4. In the $\varphi_{2}$ = 0° section, the P and Goss texture intensities were 13.9 and 10.4, respectively. In the $\varphi_{2}$ = 45° section, the P texture further increased to 15.8 and the Goss texture was 12.2. In the $\varphi_{2}$ = 65° section, a certain intensity of Cube-related orientation remained, with a maximum orientation of 15.8. These results indicate that, in the final forming region, a complex deformation texture composed of P, Goss, and residual Cube components was formed. The enhanced P and Goss components indicate pronounced local plastic flow and crystallographic reorientation. Residual Cube components nevertheless persisted locally or were redistributed during deformation.

Taken together, the pole-figure and ODF results show that the texture evolution of the Al 1060 sheet during SPIF changed from an initially strong Cube-related orientation toward multi-component textures, including Copper, S, P, Goss, and residual Cube components. With increasing forming depth, the original Cube texture gradually weakened, while deformation-related texture components became more pronounced. In region 4, the maximum texture intensity decreased and the orientation distribution became more diffuse. These results indicate that combined local stretching, bending, and shear deformation during SPIF significantly promotes grain-orientation rotation and texture reconstruction, causing different forming regions to exhibit distinct orientation-evolution characteristics [35,47].

#### 3.3.3. Misorientation Distribution

**Figure 12:**
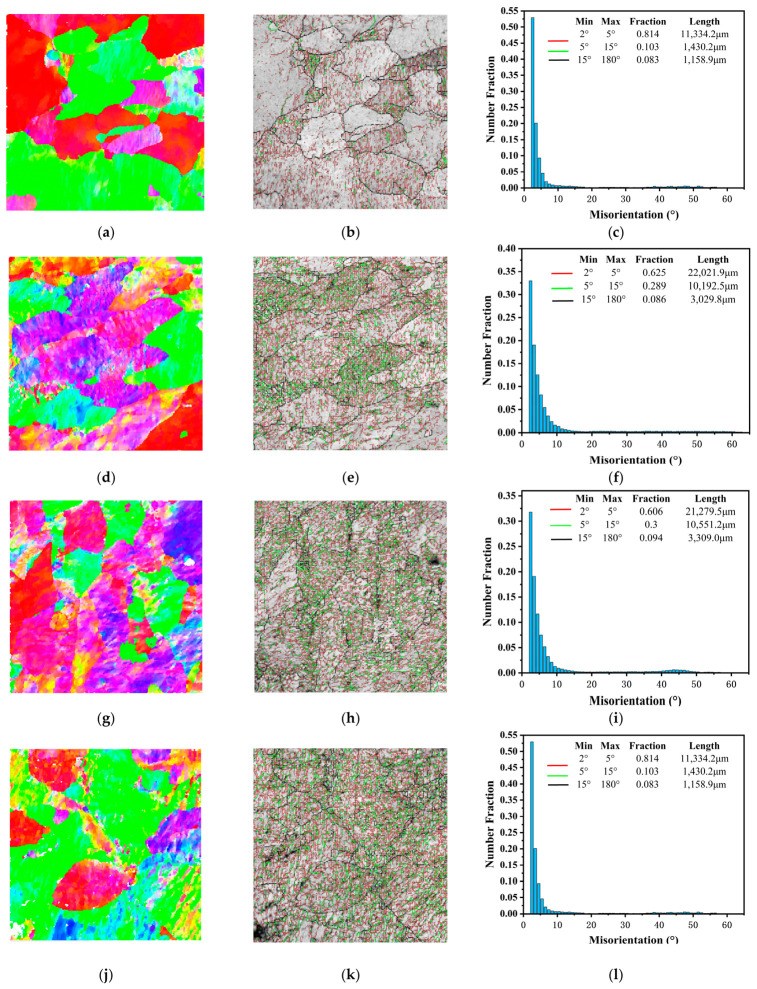
ND-IPF maps, grain-boundary maps, and misorientation-angle distributions: (**a**–**c**) region 1; (**d**–**f**) region 2; (**g**–**i**) region 3; (**j**–**l**) region 4.

The grain-boundary misorientation distribution was analyzed to evaluate intragranular orientation-gradient accumulation, substructural evolution, and grain-boundary reconstruction during SPIF. Before comparing the four regions, a common sample coordinate system was defined based on the rolling direction of the original sheet. Based on the coordinate system defined above, all specimens were mounted and analyzed using the same orientation convention. Figure 12 presents the normal-direction inverse pole figure (ND-IPF) maps, grain-boundary maps, and misorientation-angle statistics for the four regions. The colors in the misorientation-angle distributions correspond to the grain boundary types marked in the grain-boundary maps. In these maps, the color assigned to each grain in the IPF maps represents the crystallographic direction parallel to ND. Because the same RD–TD–ND coordinate system and IPF reference direction were used for all specimens, the grain-orientation distributions in regions 2–4 can be compared directly with those in the unformed reference region 1. In this study, grain boundaries were classified according to misorientation angle as low-angle grain boundaries (2–5°), medium-angle grain boundaries (5–15°), and high-angle grain boundaries (>15°).

Figure 12c shows that the fraction of low-angle grain boundaries in region 1 was as high as 81.4%, the fraction of medium-angle grain boundaries was 10.3%, and the fraction of high-angle grain boundaries was only 8.3%. This distribution indicates that the unformed reference microstructure contained numerous low-misorientation substructures, including subgrain boundaries and residual deformation structures associated with the prior rolling history of the sheet. Overall, the microstructure was dominated by low-angle grain boundaries. As illustrated in Figure 12f,i,l, as SPIF proceeded, the fraction of low-angle grain boundaries gradually decreased to 62.5%, 60.6%, and 57.3%, respectively. In contrast, the fraction of medium-angle grain boundaries increased significantly to 28.9%, 30.0%, and 34.5%, respectively. This change indicates that local plastic deformation promoted the accumulation of intragranular orientation gradients, causing the original low-angle boundaries to evolve gradually into medium-angle boundaries through continuous dislocation absorption, subgrain rotation, and increasing misorientation [45]. In particular, region 4 had the highest fraction of medium-angle grain boundaries, indicating stronger local plastic deformation and substructural reconstruction.

By comparison, the fraction of high-angle grain boundaries varied only slightly, fluctuating within the range of 8.2–9.4%, and did not increase significantly with forming depth. Under the present SPIF conditions, microstructural evolution was dominated by the transition from low- to medium-angle grain boundaries, increasing intragranular misorientation, and substructural reconstruction. The nearly unchanged high-angle grain-boundary fraction provides no clear evidence of extensive recrystallization. This behavior may be associated with the high stacking-fault energy of commercially pure Al 1060. At room temperature, dislocation cross-slip and rearrangement can release some stored deformation energy through recovery-related processes. Consequently, substructural evolution may be favored over the nucleation and growth of new high-angle grain boundaries.

The observed conversion from low-angle to medium-angle grain boundaries is consistent with the progressive rotation and subdivision of deformation-induced substructures. Similar changes in grain-boundary misorientation and deformation substructure have been reported during SPIF of AA1050 sheets, where increasing forming strain promoted grain fragmentation, intragranular orientation gradients, and the development of deformation-induced boundaries [9,10]. However, because the high-angle grain-boundary fraction remained nearly unchanged in the present study, the EBSD results do not provide clear evidence of extensive recrystallization. Instead, they are more consistent with progressive subgrain rotation and dislocation rearrangement during localized plastic deformation.

In summary, the grain-boundary misorientation results show that SPIF significantly changed the grain-boundary structure of the Al 1060 sheet. With increasing forming depth, the fraction of low-angle grain boundaries gradually decreased, the fraction of medium-angle grain boundaries increased significantly, and the fraction of high-angle grain boundaries remained relatively stable. This indicates that local plastic strain accumulation during forming promoted dislocation rearrangement, subgrain rotation, and increasing grain-boundary misorientation, providing experimental evidence for subsequent KAM, GND density distribution analysis and macro–micro correlation discussion.

#### 3.3.4. KAM and GND Density Characterization

To assess the spatial heterogeneity of plastic deformation during SPIF, kernel average misorientation (KAM) was calculated as the average misorientation between each indexed EBSD point and its neighboring points within the same grain. Neighboring-point pairs with misorientations greater than 5° were excluded to minimize the contribution of grain boundaries. KAM is sensitive to local lattice rotation and intragranular orientation gradients. It is therefore widely used as a qualitative or semi-quantitative indicator of heterogeneous plastic deformation rather than as a direct measurement of plastic strain [13,14,48].

Geometrically necessary dislocations (GNDs) accommodate non-uniform plastic deformation and the associated lattice curvature. Their density can therefore be estimated from EBSD-derived orientation gradients using the Nye-tensor framework or related local-misorientation approximations [15,49]. In the present study, the local GND density was estimated from the KAM value using Equation (8):

(8) $$\rho_{\mathrm{GND}}=\frac{2\rho_{\mathrm{KAM}}}{b\times s}$$

where $\rho_{\mathrm{GND}}$ is the GND density, $\rho_{\mathrm{KAM}}$ is the average KAM value in radians, b is the magnitude of the Burgers vector taken as 0.286 nm, and s is the EBSD step size taken as 1.0 μm. These parameters are consistent with the EBSD acquisition parameters mentioned above.

The GND density obtained from conventional two-dimensional EBSD represents a lower-bound estimate of the dislocation content associated with measurable lattice curvature rather than the total dislocation density. The calculated value is sensitive to the EBSD step size, angular resolution, neighboring-point definition, and data-cleaning procedure [15]. Jiang et al. [50] demonstrated that both the recovered GND density and its measurement noise floor depend on the EBSD step size and orientation uncertainty. Therefore, identical acquisition and post-processing parameters, including a step size of 1.0 μm, were applied to all four regions. The resulting GND values were used primarily for regional comparison rather than as absolute measurements of the total dislocation density.

**Figure 13:**
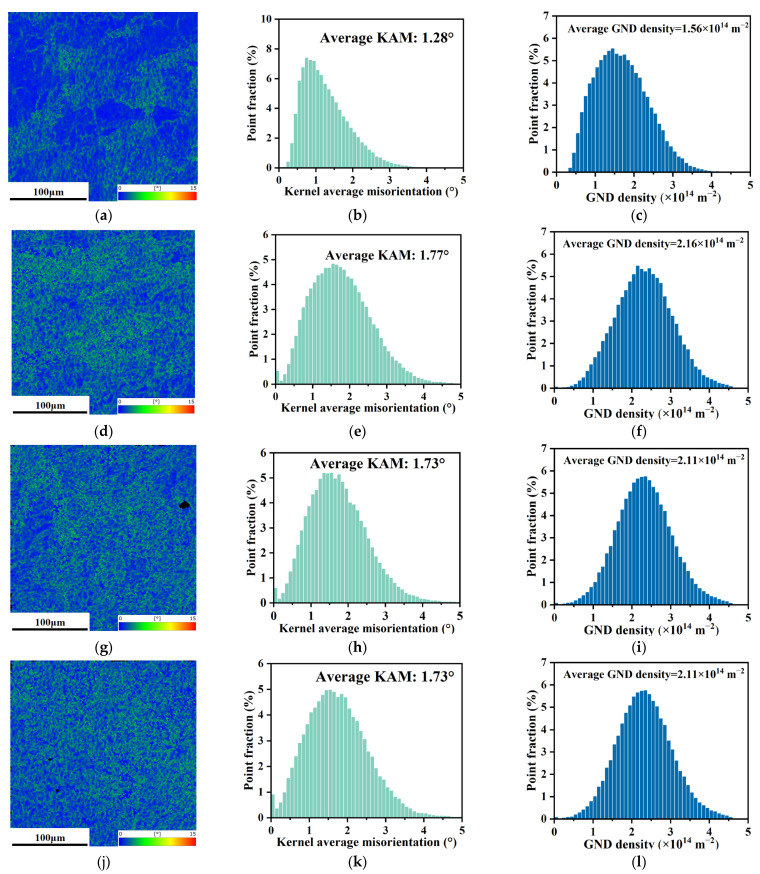
KAM maps, KAM distributions, and corresponding GND-density distributions: (**a**–**c**) region 1; (**d**–**f**) region 2; (**g**–**i**) region 3; (**j**–**l**) region 4.

Figure 13 presents the KAM maps, KAM distributions, and corresponding GND density distributions for the four regions. The black pixels in Figure 13a,d,g,j represent non-indexed EBSD points. These points were mainly associated with poor local diffraction-pattern quality, potentially resulting from severe deformation, surface-preparation damage, or local surface relief. Because no reliable crystallographic orientation could be assigned to these pixels, they were excluded from the KAM and GND density calculations and the corresponding point-fraction statistics.

As illustrated in Figure 13b,c, the unformed reference region, region 1, exhibited an average KAM of 1.28 ± 0.05° and an average GND density of (1.56 ± 0.08) ×10^14^ m^−2^. Both distributions were relatively narrow and concentrated at lower values. The non-zero baseline values should not be attributed to SPIF deformation because region 1 was located outside the tool path and had a predicted PEEQ value of zero. Instead, they may reflect orientation gradients and stored dislocations inherited from the rolling history of the as-received sheet, together with the finite angular resolution-dependent noise floor of conventional EBSD. Region 1 therefore provided an experimental reference for evaluating the additional orientation gradients introduced by SPIF.

In region 2, the average KAM increased from 1.28 ± 0.05° to 1.77 ± 0.08°, corresponding to an increase of approximately 38.3%. The average GND density similarly increased from (1.56 ± 0.08) ×10^14^ to (2.16 ± 0.13) ×10^14^ m^−2^, an increase of approximately 38.5%. The simultaneous rightward shifts of the KAM and GND density distributions indicate that the initial tool–sheet interaction generated pronounced intragranular orientation gradients and spatially heterogeneous plastic deformation. This interpretation is consistent with the role of GNDs in accommodating lattice curvature associated with non-uniform plastic deformation [51].

The observed trend is also consistent with previous EBSD investigations of commercially pure aluminum subjected to incremental forming. Shrivastava and Tandon [9] reported increasing intragranular misorientation and deformation-induced microstructural subdivision at higher SPIF strain levels. Kumar et al. [51] similarly observed increasing KAM and GND density with increasing wall angle in AA1050, with pronounced GND accumulation in strongly localized regions near failure. Although their forming conditions and strain levels differed from those employed in the present study, the direction of the microstructural evolution was consistent.

As shown in Figure 13h,i,k,l, the average KAM stabilized at approximately 1.73 ± 0.07° in region 3 and 1.73 ± 0.07° in region 4. The corresponding average GND density remained at approximately (2.11 ± 0.11) ×10^14^ m^−2^ and (2.11 ± 0.14) ×10^14^ m^−2^ respectively. This plateau does not indicate that plastic deformation or dislocation storage ceased. KAM-derived GND density reflects the net dislocation content associated with orientation gradients at the selected EBSD length scale. Additional dislocations may be stored as statistically stored dislocations (SSDs) without producing a proportional increase in lattice curvature. Existing dislocations may also be redistributed into deformation-induced substructures. The nearly constant KAM and GND density values therefore indicate that the measurable orientation gradients approached a quasi-stable stage after their rapid development in region 2.

Aluminum has a relatively high stacking-fault energy, which facilitates dislocation cross-slip and rearrangement during deformation [30]. Recovery-like rearrangement may therefore have contributed to the stabilization of the local orientation gradients. However, the present EBSD measurements do not independently demonstrate dynamic recovery. The plateau is more cautiously interpreted as being consistent with the combined effects of continued dislocation storage, rearrangement, and substructure development. This interpretation is also supported by the increase in the medium-angle grain-boundary fraction without a pronounced increase in the high-angle grain-boundary fraction. Thus, the current EBSD results provide no clear evidence of extensive recrystallization.

It should also be emphasized that the KAM-derived GND density is not directly equivalent to the SSD density inferred from the FE-predicted PEEQ field using the K–M model. The former represents the net dislocation content associated with measurable lattice curvature, whereas the latter represents statistically stored dislocations predicted from strain accumulation. The two quantities were therefore compared only in terms of their regional evolution rather than their absolute magnitudes.

Overall, the KAM and GND density results revealed a pronounced increase in deformation heterogeneity from region 1 to region 2, followed by the stabilization of measurable orientation gradients in regions 3 and 4. This stage-wise evolution agrees qualitatively with previous EBSD investigations of incrementally formed AA1050, which reported increasing local misorientation, grain subdivision, and GND accumulation with increasing forming severity [9,10]. In the present study, the most pronounced increase in orientation-gradient-related deformation occurred during the initial tool-affected stage. Subsequent deformation coincided with further grain refinement, redistribution of grain-boundary misorientation, and continued K–M-inferred SSD accumulation.

### 3.4. Analysis of Correlation Mechanism Between Macroscopic Forming Quality and Microstructural Response

To clarify the relationship between macroscopic forming quality, FE-inferred SSD density, and EBSD-derived microstructural evolution, a regional quantitative analysis was performed on the formed part. To correlate with the EBSD characterization, the SSD density was inferred from the FE PEEQ result at the final forming stage through the K–M model. Due to the spatially varying deformation history in SPIF, the regional SSD density distribution at the final forming stage still reflects the cumulative evolution of plastic deformation and dislocation storage. The regional quantitative comparison between macroscopic forming quality, K–M-inferred SSD density, and EBSD-derived microstructural indicators is summarized in Table 3. As illustrated in Table 3, the average thinning rate increased from 0% (region 1) to 6.795% (region 2), 13.929% (region 3), and 15.910% (region 4), indicating progressive thickness reduction. In parallel, the SSD density increased from 0.450 × 10^13^ m^−2^ to 1.420 × 10^13^ m^−2^ and then stabilized at 1.550 × 10^13^ m^−2^, suggesting saturation of dislocation storage at higher deformation levels. Consistently, EBSD results showed continuous grain refinement from 30.4 μm to 21.9 μm and an increase in the medium-angle grain-boundary (MAGB) fraction from 10.3% to 34.5%, indicating progressive substructure formation and boundary reconstruction driven by accumulated plastic strain.

**Figure 14:**
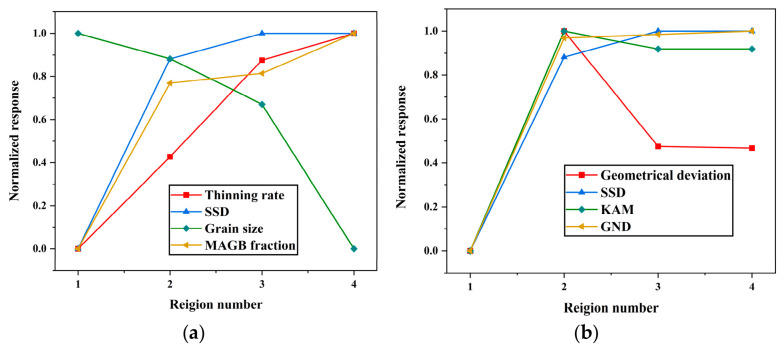
Normalized regional relationships between forming quality and microstructural indicators: (**a**) thinning rate and (**b**) geometrical deviation.

Figure 14 shows the normalized regional correlations between macroscopic forming quality indicators and microstructural indicators, thereby providing a direct comparison of the mechanisms associated with thickness reduction and geometrical deviation. As shown in Figure 14a, the thinning rate increases with the SSD density and MAGB fraction, accompanied by a gradual reduction in grain size. This correlation indicates that thickness reduction is closely associated with cumulative plastic deformation, dislocation storage, and deformation-induced substructure evolution. In particular, the increase in SSD density reflects enhanced dislocation accumulation during incremental forming, whereas the increase in MAGB fraction and the reduction in grain size suggest the development of subgrain structures and grain refinement under accumulated plastic strain.

Figure 14b compares geometrical deviation with SSD density, KAM, and GND density, showing that the geometrical deviation does not increase monotonically during SPIF. Region 1 remains unformed, exhibiting low SSD density, KAM, and GND density together with a relatively coarse-grained structure. In region 2, the geometrical deviation reaches its maximum, accompanied by sharp increases in SSD density, KAM, and GND density. This indicates that the initial formation of geometrical deviation is closely related to deformation localization and the rapid development of orientation gradients during the early forming stage. Continued strain accumulation in regions 3 and 4 promoted further grain refinement and increased the MAGB fraction. However, KAM and GND density remained nearly constant, suggesting that measurable orientation gradients approached a plateau after the early deformation stage. Meanwhile, the geometrical deviation decreases slightly. Therefore, the geometrical deviation in SPIF is closely related to deformation localization at the early stage, which is accompanied by the rapid accumulation of orientation gradients and GND density.

**Figure 15:**
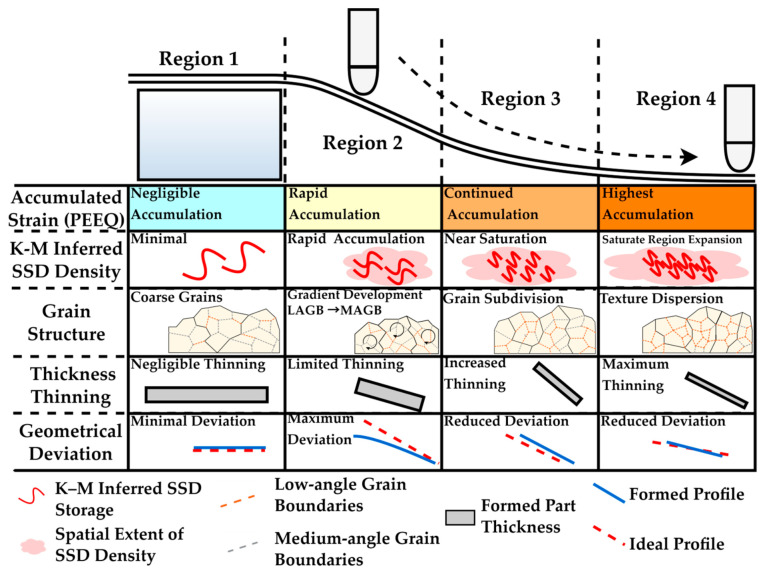
Schematic illustration of the proposed stage-dependent deformation mechanisms across regions 1–4 during SPIF.

Figure 15 summarizes the proposed deformation mechanisms across regions 1–4. Initial tool loading in region 2 produces rapid PEEQ and K–M-inferred SSD accumulation, accompanied by the development of intragranular orientation gradients and the maximum geometrical deviation. In regions 3 and 4, PEEQ continues to increase, whereas the local inferred SSD density approaches saturation and the high-SSD density region expands spatially. Continued deformation promotes grain subdivision, the transition from low- to medium-angle grain boundaries, grain refinement, and texture dispersion. The plateaus in KAM and GND density indicate stabilization of measurable orientation gradients rather than cessation of plastic deformation. These stage-dependent responses explain why thinning increases with cumulative deformation, whereas geometrical deviation is more sensitive to early deformation localization.

The observed microstructural evolution can be interpreted as a sequential response to repeated localized loading during SPIF. At the early tool-affected stage, rapid plastic strain accumulation promotes dislocation multiplication and the development of intragranular orientation gradients, as reflected by the sharp increases in KAM and GND density. With continued deformation, dislocation rearrangement and subgrain rotation promote the transition from low- to medium-angle boundaries and contribute to the measured grain refinement. Meanwhile, the weakening of the initial Cube texture and the emergence of multiple deformation-related texture components are consistent with grain rotation under the combined stretching, bending, and shear deformation imposed by successive tool passes. The K–M-inferred SSD saturation reflects the competition between dislocation storage and recovery in the model. Therefore, the subsequent plateau in KAM and GND density should be interpreted as stabilization of measurable orientation gradients at the selected EBSD length scale rather than cessation of plastic deformation or complete microstructural stabilization. Previous studies of AA1050 have shown that repeated localized tool contact can lead to heterogeneous strain accumulation, intragranular lattice rotation, grain fragmentation, and increased dislocation density [9,10]. From region 1 to region 2, the rapid increases in PEEQ, inferred SSD density, KAM, and GND density indicate the concurrent development of plastic strain accumulation, dislocation storage, and local strain gradients during the early stage of deformation. From region 2 to region 4, PEEQ and inferred SSD density continued to increase, whereas KAM and GND density increased more gradually and tended to approach a plateau. This divergence may arise because further deformation promotes the accumulation and interaction of statistically stored dislocations without necessarily producing an equivalent increase in the net lattice curvature detected by EBSD. Thus, the inferred SSD density may continue to evolve even when KAM and GND density become relatively stable. Nevertheless, these observations represent spatial and stage-wise correlations among the FE-derived and EBSD-derived indicators rather than direct causal verification. Confirmation of the proposed mechanism would require interrupted forming experiments, higher-resolution EBSD, TEM characterization, or crystal-plasticity analysis.

Direct experimental measurements of SSD density were not conducted in the present study; therefore, the EBSD-derived KAM and GND-density results provide only indirect cross-scale support for the predicted SSD evolution. Because these EBSD indicators reflect local lattice curvature and strain gradient-related dislocations rather than SSD density itself, their agreement should not be interpreted as quantitative validation of the absolute SSD values.

Overall, the results support a cross-scale relationship between forming quality and microstructural evolution during SPIF of Al 1060. Thickness reduction was associated primarily with accumulated plastic strain and dislocation storage. Geometrical deviation was more sensitive to early-stage deformation heterogeneity and orientation-gradient accumulation.

## 4. Conclusions and Future Work

### 4.1. Conclusions

This study developed a K–M-based macro–micro correlation framework integrating forming experiments, FE simulation, SSD density inference, and EBSD characterization to investigate the relationship between forming quality and microstructural response during SPIF of Al 1060. The main conclusions and scientific implications are as follows:

(1) The proposed framework provides a computationally efficient route for linking process-scale plastic deformation with dislocation-based microstructural evolution. The K–M-inferred SSD density approached a saturation value of approximately 1.55 × 10^13^ m^−2^ during the early forming stage, while the high-SSD density region continued to expand during subsequent forming. This distinction shows that later-stage microstructural evolution was governed primarily by the spatial extension of elevated dislocation storage rather than by a continued increase in the local peak value.
(2) EBSD characterization revealed progressive microstructural reconstruction across the forming regions. The average grain size decreased from 30.4 ± 2.1 μm to 21.9 ± 1.4 μm, while the medium-angle grain-boundary fraction increased from 10.3% to 34.5%. These changes indicate that repeated localized loading promoted grain subdivision, subgrain rotation, and the progressive transition from low- to medium-angle boundaries.
(3) By comparing the geometrical deviation, thinning rate, FE-derived PEEQ, K-M-inferred SSD density, and EBSD microstructural indicators, the macro–micro correlation mechanism of SPIF forming quality was clarified. Thickness reduction increased progressively with PEEQ and the K–M-inferred SSD density, indicating its close relationship with cumulative plastic deformation and dislocation storage. In contrast, the maximum geometrical deviation occurred in the early tool-affected region together with rapid increases in KAM and GND density. This distinction suggests that thinning and geometrical accuracy arise from different stages of the deformation history and may therefore require different process-control strategies.

### 4.2. Future Work

Although this study established a macro–micro correlation framework for SPIF of Al 1060 sheets, several aspects require further investigation:

(1) This work focused on a truncated-cone part under a single process-parameter combination. Future studies should examine the effects of tool radius, vertical step size, wall angle, feed rate, and forming path on thinning, geometrical deviation, PEEQ and SSD density distribution, and microstructural response.
(2) Future work will employ complementary characterization techniques, such as transmission electron microscopy and X-ray line-profile analysis, to quantify dislocation structures and provide a more direct assessment of the K–M-inferred SSD density. Higher-resolution EBSD and crystal-plasticity simulations may further clarify the relationships between SSD storage, GND accumulation, slip-system activity, and local orientation gradients.
(3) This study mainly compared microstructural differences among regions of the final formed part. Staged interruption experiments and sampling at different forming depths would help reveal the continuous evolution of grain size, grain-boundary misorientation, texture, KAM, GND density, and SSD density during forming.
(4) These correlations may guide future process optimization. Regions with high PEEQ, high inferred SSD density, pronounced thinning, or large geometrical deviations may serve as reference indicators for tool-path design and parameter selection.
(5) The present microstructural analysis was based on four representative regions corresponding to the principal deformation stages. Although this design captures the main stage-wise trends, it does not fully resolve continuous spatial gradients or localized transitions between adjacent regions. Future studies will employ more densely spaced sampling locations along the forming path to improve spatial resolution and characterize the continuous evolution of grain structure, crystallographic orientation, KAM, and dislocation density.

## Figures and Tables

**Table 1:** PEEQ statistics at different forming stages.

Forming Stage	Maximum PEEQ	Average PEEQ	PEEQ > 0.5 (%)	PEEQ > 1.0 (%)
Unformed	0.000	0.000	0.000	0.000
Initial forming	0.965	0.069	3.740	0.000
Intermediate forming	1.110	0.116	10.110	1.120
Late forming	1.425	0.154	13.610	5.530
Final forming	1.760	0.175	14.980	7.530

**Table 2:** K–M-inferred SSD statistics at different forming stages.

Forming Stage	Maximum SSD Density (m^−2^)	Average SSD Density (m^−2^)	SSD Density >1.0 × 10^13^ (%)	SSD Density >1.5 × 10^13^ (%)
Unformed	1.00 × 10^12^	1.00 × 10^12^	0.00	0.00
Initial forming	1.53 × 10^13^	4.39 × 10^12^	15.19	0.66
Intermediate forming	1.53 × 10^13^	4.60 × 10^12^	19.04	6.78
Late forming	1.55 × 10^13^	4.68 × 10^12^	20.87	10.63
Final forming	1.55 × 10^13^	4.75 × 10^12^	21.26	12.19

**Table 3:** Regional quantitative comparison between macroscopic forming quality, K–M-inferred SSD density, and EBSD-derived microstructural indicators.

Region	Average Geometrical Deviation (mm)	Average Thinning Rate (%)	Average K–M-Inferred SSD Density (×10^13^ m^−2^)	Average Grain Size (μm)	MAGB Fraction (%)	Average KAM (°)	Average GND Density (×10^14^ m^−2^)
Region 1	0.000	0.000	0.450	30.400	10.300	1.280	1.560
Region 2	2.324	6.795	1.420	29.400	28.900	1.770	2.160
Region 3	1.104	13.929	1.550	27.600	30.000	1.730	2.110
Region 4	1.086	15.910	1.550	21.900	34.500	1.730	2.110

## Data Availability

The original contributions presented in this study are included in the article. Further inquiries can be directed to the corresponding authors.

## Abbreviations

The following abbreviations are used in this manuscript:

EBSD	Electron backscatter diffraction
FCC	Face-centered cubic
FE	Finite element
GND	Geometrically necessary dislocation
HAGB	High-angle grain boundary
IPF	Inverse pole figure
KAM	Kernel average misorientation
K–M	Kocks–Mecking
LAGB	Low-angle grain boundary
MAGB	Medium-angle grain boundary
ND	Normal direction
ODF	Orientation distribution function
OM	Optical microscopy
PEEQ	Equivalent plastic strain
RD	Rolling direction
SEM	Scanning electron microscopy
SPIF	Single-point incremental forming
SSD	Statistically stored dislocation
TD	Transverse direction
TEM	Transmission electron microscopy
VPSC	Viscoplastic self-consistent
WEDM	Wire electrical discharge machining
**Symbols**	
σ	True stress in the strain-hardening regime
$\sigma_{s}$	Saturation flow stress
$\varepsilon_{p}$	Equivalent plastic strain corresponding to PEEQ
A	Voce hardening parameter controlling the initial offset from saturation stress
B	Voce hardening parameter controlling the saturation rate
ρ	SSD density
b	Magnitude of the Burgers vector
γ	Equivalent shear strain
M	Taylor factor
$\rho_{0}$	Initial SSD density
α	Dislocation interaction coefficient
G	Shear modulus
a	Lattice parameter of FCC aluminum
$\rho_{\mathrm{sat}}$	Saturated SSD density
$\rho_{\mathrm{GND}}$	GND density
$\rho_{\mathrm{KAM}}$	Average KAM value in radians

## References

[1] Jeswiet J., Micari F., Hirt G., Bramley A., Duflou J., Allwood J. (2005). Asymmetric single point incremental forming of sheet metal. CIRP Ann..

[2] Duflou J.R., Habraken A.-M., Cao J., Malhotra R., Bambach M., Adams D., Vanhove H., Mohammadi A., Jeswiet J. (2018). Single point incremental forming: State-of-the-art and prospects. Int. J. Mater. Form..

[3] Fang Y., Lu B., Chen J., Xu D., Ou H. (2014). Analytical and experimental investigations on deformation mechanism and fracture behavior in single point incremental forming. J. Mater. Process. Technol..

[4] Yazar K.U., Mishra S., Narasimhan K., Date P.P. (2019). Deciphering the deformation mechanism in single point incremental forming: Experimental and numerical investigation. Int. J. Adv. Manuf. Technol..

[5] Micari F., Ambrogio G., Filice L. (2007). Shape and dimensional accuracy in single point incremental forming: State of the art and future trends. J. Mater. Process. Technol..

[6] Lu H., Liu H., Wang C. (2019). Review on strategies for geometric accuracy improvement in incremental sheet forming. Int. J. Adv. Manuf. Technol..

[7] Zhu X., Zhang X., Jiang J., Wu X., Liao S., Huang J., Wang Y. (2026). An improved multi-objective grey wolf optimizer for bi-objective parameter optimization in single point incremental forming of Al1060 sheet. Materials.

[8] Barnwal V.K., Chakrabarty S., Tewari A., Narasimhan K., Mishra S.K. (2018). Forming behavior and microstructural evolution during single point incremental forming process of AA-6061 aluminum alloy sheet. Int. J. Adv. Manuf. Technol..

[9] Shrivastava P., Tandon P. (2019). Microstructure and texture based analysis of forming behavior and deformation mechanism of AA1050 sheet during single point incremental forming. J. Mater. Process. Technol..

[10] Kumar A., Shrivastava A., Narasimhan K., Mishra S. (2022). Microstructure and texture evolution during incremental sheet forming of AA1050 alloy. J. Mater. Sci..

[11] Hussain G., Ilyas M., Lemopi Isidore B., Khan W.A. (2020). Mechanical properties and microstructure evolution in incremental forming of AA5754 and AA6061 aluminum alloys. Trans. Nonferrous Met. Soc. China.

[12] Ghaferi M., Mirnia M.J., Elyasi M., Jamshidi Aval H. (2019). Evaluation of different heat treatment cycles on improving single point incremental forming of AA6061 aluminum alloy. Int. J. Adv. Manuf. Technol..

[13] Wright S.I., Nowell M.M., Field D.P. (2011). A review of strain analysis using electron backscatter diffraction. Microsc. Microanal..

[14] Kamaya M., Wilkinson A.J., Titchmarsh J.M. (2005). Measurement of plastic strain of polycrystalline material by electron backscatter diffraction. Nucl. Eng. Des..

[15] Konijnenberg P., Zaefferer S., Raabe D. (2015). Assessment of geometrically necessary dislocation levels derived by 3D EBSD. Acta Mater..

[16] Baudin T., Azzeddine H., Brisset F., Huang Y., Langdon T.G. (2024). Estimating dislocation density from electron backscatter diffraction data for an AZ31/Mg-0.6Gd hybrid alloy fabricated by high-pressure torsion. Philos. Mag..

[17] Roters F., Eisenlohr P., Bieler T.R., Raabe D. (2010). Crystal Plasticity Finite Element Methods in Materials Science and Engineering.

[18] Lebensohn R.A., Tomé C.N. (1993). A self-consistent anisotropic approach for the simulation of plastic deformation and texture development of polycrystals: Application to zirconium alloys. Acta Metall. Mater..

[19] Tran M.T., Lee H.-J., Phan H.C., Kang S.-H., Kim D.-K., Lee H.W. (2025). Microstructure-informed forming limits in aluminum alloys: Experiments and crystal plasticity simulations. Mater. Charact..

[20] Rakshit R., Katiyar B.S., Tomé C.N., Panda S.K., Mandal S. (2023). A finite element coupled visco-plastic self-consistent simulation to predict micro-texture and anisotropy evolution during single point incremental forming in Al-Li alloy. J. Mater. Process. Technol..

[21] Weeks J.S., Stebner A.P. (2025). Efficient multiscale simulations of incremental sheet forming using machine learning surrogate models for crystal plasticity. Integr. Mater. Manuf. Innov..

[22] Harfoush A., Tabei A., Haapala K.R., Ghamarian I. (2023). A framework for predicting grain morphology during incremental sheet metal forming using generative adversarial networks. Manuf. Lett..

[23] Zhang Y., Zhang Z., Li Y., Hu L., Pang Q., Hu Z. (2023). Investigation of Pre-Aged Hardening Single-Point Incremental Forming Process and Mechanical Properties of AA6061 Aluminum Alloy. Materials.

[24] Yang M., Chang Z., Chen J., An D. (2022). Mechanism of Dislocation Promoting Al_13_Fe_4_ Precipitation during Friction Stir-Assisted Incremental Sheet Forming in AA5052 Sheet. Mater. Lett..

[25] Kubin L., Estrin Y. (1990). Evolution of dislocation densities and the critical conditions for the Portevin–Le Châtelier effect. Acta Metall. Mater..

[26] Roters F., Raabe D., Gottstein G. (2000). Work hardening in heterogeneous alloys—A microstructural approach based on three internal state variables. Acta Mater..

[27] Ma A., Roters F., Raabe D. (2006). A dislocation density based constitutive model for crystal plasticity FEM including geometrically necessary dislocations. Acta Mater..

[28] Mecking H., Kocks U.F. (1981). Kinetics of flow and strain-hardening. Acta Metall..

[29] Estrin Y., Mecking H. (1984). A unified phenomenological description of work hardening and creep based on one-parameter models. Acta Metall..

[30] Kocks U.F., Mecking H. (2003). Physics and phenomenology of strain hardening: The FCC case. Prog. Mater. Sci..

[31] Wang Y., Zhao H., Chen X., Gault B., Bréchet Y., Hutchinson C. (2024). The effect of shearable clusters and precipitates on dynamic recovery of Al alloys. Acta Mater..

[32] Balaji V., Krishnaswamy H., Natarajan S., Lee M.-G. (2024). Modelling time-dependent relaxation behaviour using physically based constitutive framework. Int. J. Mech. Sci..

[33] Akhondzadeh S., Sills R.B., Bertin N., Cai W. (2020). Dislocation density-based plasticity model from massive discrete dislocation dynamics database. J. Mech. Phys. Solids.

[34] (2024). Standard Test Methods for Tension Testing of Metallic Materials.

[35] Shamsari M., Mirnia M.J., Elyasi M., Baseri H. (2018). Formability improvement in single point incremental forming of truncated cone using a two-stage hybrid deformation strategy. Int. J. Adv. Manuf. Technol..

[36] Taylor G.I. (1934). The mechanism of plastic deformation of crystals. Part I.—Theoretical. Proc. R. Soc. A.

[37] Mecking H., Kocks U.F., Hartig C. (1996). Taylor factors in materials with many deformation modes. Scr. Mater..

[38] Davis J.R. (1990). Properties of wrought aluminum and aluminum alloys. ASM Handbook, Volume 2: Properties and Selection: Nonferrous Alloys and Special-Purpose Materials.

[39] Nakashima P.N.H., Totten G.E., Tiryakioglu M., Kessler O. (2019). The crystallography of aluminum and its alloys. The Encyclopedia of Aluminum and Its Alloys.

[40] Bukvić M., Vencl A., Milojević S., Skulić A., Gajević S., Stojanović B. (2025). The Influence of Carbon Nanotube Additives on the Efficiency and Vibrations of Worm Gears. Lubricants.

[41] Hoang T.-K., Luyen T.-T., Nguyen D.-T. (2023). Enhancing/Improving Forming Limit Curve and Fracture Height Predictions in the Single-Point Incremental Forming of Al1050 Sheet Material. Materials.

[42] Pepelnjak T., Sevšek L., Lužanin O., Milutinović M. (2022). Finite Element Simplifications and Simulation Reliability in Single Point Incremental Forming. Materials.

[43] Al-Ghamdi K.A., Hussain G. (2015). The Pillowing Tendency of Materials in Single-Point Incremental Forming: Experimental and Finite Element Analyses. Proc. Inst. Mech. Eng. Part B J. Eng. Manuf..

[44] Ge T., Chu X., Liu C., Yue Z., Li Y. (2024). A cryogenic incremental sheet forming process for improving the formability of AA6061 to reveal the dual enhancement effect and microstructure evolution mechanism. J. Mater. Res. Technol..

[45] Muñoz J.A., de Castro A., Albo K., Jiménez-Piqué E., Ortiz Membrado L., Sánchez-Soto M., Cabrera J.M. (2024). Heterostructured pure aluminum produced by impact loading. Mater. Sci. Eng. A.

[46] Engler O., Randle V. (2010). Introduction to Texture Analysis: Macrotexture, Microtexture, and Orientation Mapping.

[47] Snopiński P., Hilšer O. (2024). Mechanism of grain refinement in 3D-printed AlSi10Mg alloy subjected to severe plastic deformation. Materials.

[48] Kamaya M. (2011). Assessment of local deformation using EBSD: Quantification of accuracy of measurement and definition of local gradient. Ultramicroscopy.

[49] Pantleon W. (2008). Resolving the geometrically necessary dislocation content by conventional electron backscattering diffraction. Scr. Mater..

[50] Jiang J., Britton T.B., Wilkinson A.J. (2013). Measurement of geometrically necessary dislocation density with high resolution electron backscatter diffraction: Effects of detector binning and step size. Ultramicroscopy.

[51] Kumar A., Mehtani H.K., Shrivastava A., Mishra S., Narasimhan K., Samajdar I. (2023). Failure mechanism during incremental sheet forming of a commercial purity aluminum alloy. Eng. Fail. Anal..

